# Treadmill Exercise Attenuates Cerebral Ischemia–Reperfusion Injury by Promoting Activation of M2 Microglia via Upregulation of Interleukin-4

**DOI:** 10.3389/fcvm.2021.735485

**Published:** 2021-10-04

**Authors:** Juanjuan Lu, Jie Wang, Long Yu, Rong Cui, Ying Zhang, Hanqing Ding, Guofeng Yan

**Affiliations:** ^1^Department of Rehabilitation, Shanghai Xuhui Central Hospital, Shanghai, China; ^2^School of Kinesiology, Shanghai University of Sport, Shanghai, China; ^3^Academy of Integrative Medicine, Shanghai University of Traditional Chinese Medicine, Shanghai, China; ^4^School of Medicine, Shanghai Jiao Tong University, Shanghai, China

**Keywords:** treadmill exercise, anti-inflammation, cerebral ischemia–reperfusion injury, M2 microglia, interleukin-4

## Abstract

**Background:** Exercise has been proven to be an effective therapy for stroke by reducing the microglia-initiated proinflammatory response. Few studies, however, have focused on the phenotypic changes in microglia cells caused by exercise training. The present study was designed to evaluate the influence of treadmill exercise on microglia polarization and the molecular mechanisms involved.

**Methods:** Male Sprague-Dawley rats were randomly assigned into 3 groups: sham, MCAO and exercise. The middle cerebral artery occlusion (MCAO) and exercise groups received MCAO surgery and the sham group a sham operation. The exercise group also underwent treadmill exercise after the surgery. These groups were studied after 4 and 7 days to evaluate behavioral performance using a modified neurological severity score (mNSS), and infarct conditions using 2,3,5-triphenyl tetrazolium chloride. Quantitative real-time polymerase chain reaction (qRT-PCR) and Luminex was employed to determine the expressions of markers of microglia phenotypes. Western blotting was employed to identify the phosphorylation levels of Janus kinase1 (JAK1) and signal transducer and activator of transcription 6 (STAT6). Immunofluorescence was conducted to identify microglia phenotypes.

**Results:** Treadmill exercise was found to improve neurobehavioral outcomes, mainly motor and balance functions, reduce infarct volumes and significantly increase interleukin-4 (IL-4) expression. In addition, treadmill exercise inhibited M1 microglia and promoted M2 microglia activation as evidenced by decreased M1 and increased M2 markers. Furthermore, an obvious increase in p-JAK1 and p-STAT6 was observed in the exercise group.

**Conclusions:** Treadmill exercise ameliorates cerebral ischemia–reperfusion injury by enhancing IL-4 expression to promote M2 microglia polarization, possibly via the JAK1-STAT6 pathway.

## Introduction

Clinically, early exercise has been proven to be an effective method to promote motor recovery in patients with ischemic stroke (1, 2). Experimental studies have also demonstrated that treadmill training is beneficial in ameliorating neurological deficits in rats after cerebral ischemia (3, 4). Further analysis of the mechanism of exercise-induced neuroprotection revealed decreased activation of resident cells such as microglia and astrocytes (5, 6), suppressed expression of pro-inflammatory cytokines (6, 7), and reduced leukocyte infiltration in the penumbra (8, 9), results consistent with our previous findings (10).

It has been shown that activated microglia become rapidly polarized into different phenotypes in response to ischemic conditions (11). The anti-inflammatory phenotype, known as M2, emerged at the border of the ischemic area from the 1 day, reached a peak expression at 5 days after MCAO, then decreased over 7 to 14 days after MCAO, but was still higher than in the sham group. By comparison, the inflammatory phenotype M1, appeared in the penumbra after 3 days and was maintained at a high level until day 14 after MCAO (12). These data suggested that the M1 phenotype gradually took the place of the M2 phenotype, dominating at the infarct border. Therefore, maintaining the M2 phenotype and inhibiting the M1 phenotype in the penumbra is crucial for the attenuation of cerebral ischemia reperfusion (CI/RP) injury. Our previous study found that treadmill exercise effectively inhibited microglia activation and diminished the expression of pro-inflammatory mediators, including interleukin-1β (IL-1β) and monocyte chemotactic protein-1 (MCP-1), at 3 and 6 days post-injury (10). Since the M1 phenotype is able to produce pro-inflammatory cytokines, it is reasonable to link the exercise-induced neuroprotection to a significant decline in the M1 phenotype in the penumbra. Even so, the effects of treadmill exercise on anti-inflammatory cytokines and the M2 microglia associated with these cytokines still remains unknown.

To date, IL-4 has been reported to participate in neuroprotection against cerebral ischemia and that the upregulation of M2 microglia induced by IL-4 treatment has a neuroprotective effect (13, 14). Liu et al. reported that IL-4 deficiency disrupted neurological functions and impaired microglia M2 polarization in mice after stroke (15). These investigations suggested that IL-4 is actively involved in promoting functional recovery through induction of the M2 phenotype. Recently, testing with a customized magnetic rat premixed multi analyte kits for multiple anti-inflammatory cytokines, including interleukin-10 (IL-10), interleukin-4 (IL-4), interleukin-13 (IL-13), and so on, we discovered a significant increase in the protein level of IL-4 after 3- and 6-days of treadmill exercise. We speculated that IL-4 might be involved in the process by which treadmill exercise exerts a beneficial effect after CI/RP injury. Thus, this question deserves further study of the anti-inflammatory mechanisms involved in treadmill training after MCAO and in particular the potential role of IL-4.

The Janus kinase (JAK) signal transducer and activator of transcription 6 (STAT6) signaling is acknowledged as the primary pathway involved in IL-4 responsiveness and its effects in modulating inflammatory events (16–18). Koh et al. discovered that IL-4 activated STAT6 to regulate M1/M2 polarization in various diseases arising as a result of inflammation (19). He et al. found that the administration of IL-4 led to a transition between M1 and M2a phenotypes through the JAK1/STAT6 pathway to promote post stroke recovery (20). Therefore, elucidation of the IL-4 and related signaling pathways involved in the protective effect induced by treadmill exercise in a stroke model is needed.

In the present study, we evaluated the mechanisms underlying the improvements in neurobehavioral deficits after treadmill training and whether IL-4 boosted microglia to polarize from the M1 to M2 phenotype in exercise-mediated neuroprotection in MCAO rats.

## Materials and Methods

### Animals

Adult, male Sprague Dawley (weighing 250–320 g) rats were obtained from the Slack Experimental Animal Company (Shanghai, China). The rats were housed in cages (4 rats per cage) at 23°C in a 12/12 h light/dark cycle, with *ad libitum* access to food and water. In total 117 rats were familiarized with the environment for 1 week prior to operations and then were randomly allocated into a treadmill exercise, MCAO or sham operation group. All the experimental procedures were carried out according to the National Institutes of Health (NIH) guidelines for the care and use of laboratory animals. The experimental protocols were approved by the Ethics Committee of Shanghai Jiao Tong University (Ethical code: 2019027). All efforts were made to minimize the number of animals used and their suffering.

### Middle Cerebral Artery Occlusion Model

**Figure 1:**
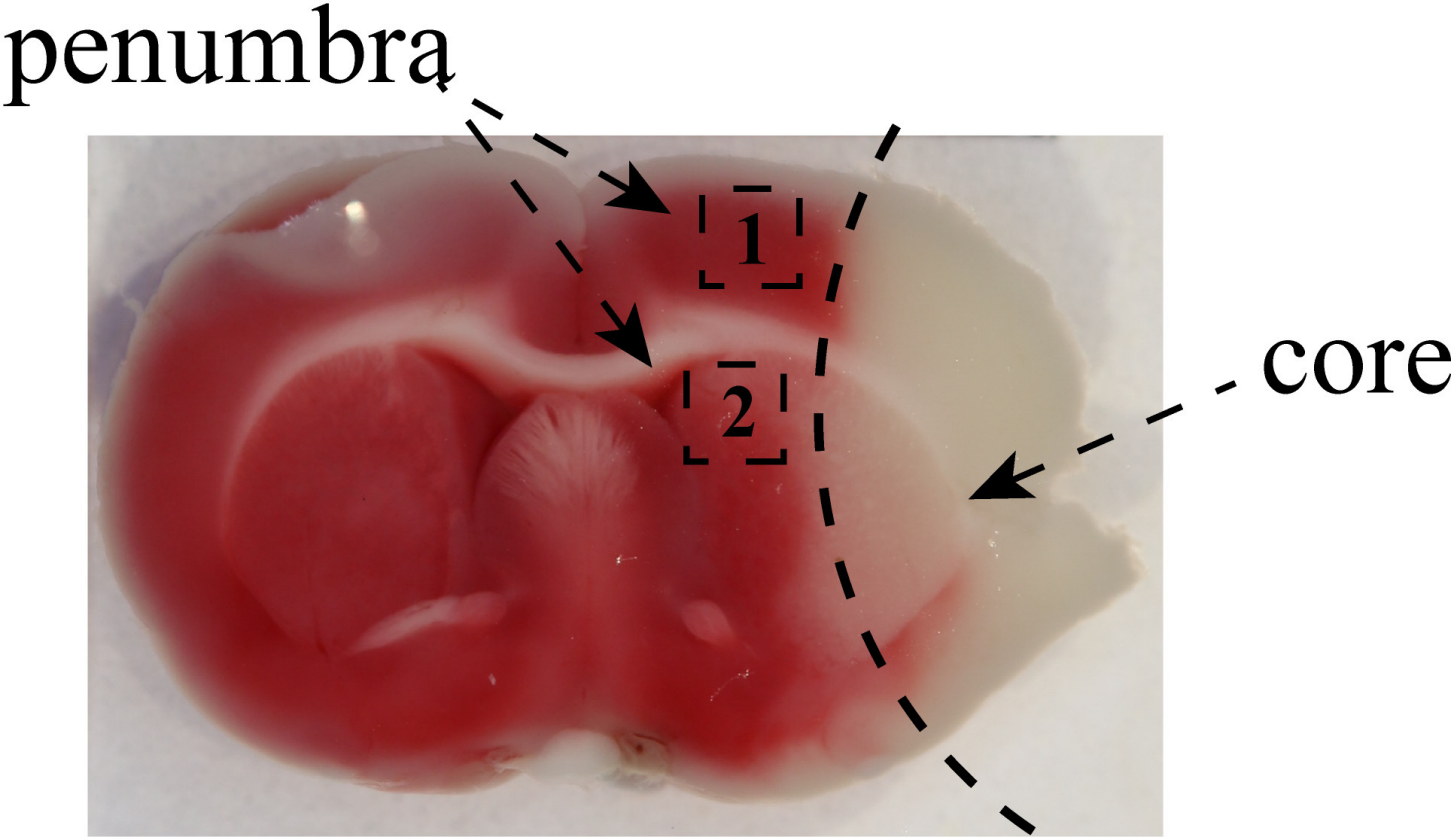
The images of the penumbra after MCAO. The images showed that the black zone marked as “1” represented the selected cortex region near the infarct core and the area labeled as “2” was marginal striatum around the infarct core. We used the brain tissue from penumbra to perform qRT-PCR, Luminex multiplex assay, and Western blotting. The area of penumbra in the cortex and striatum for immunofluorescence staining was chosen as shown.

Rats in the exercise and MCAO groups underwent right middle cerebral artery occlusion-reperfusion surgery. After accurate weighing, rats were anesthetized intraperitoneally before surgery. Subsequently, they were position on the operating table in a supine position and shaved to expose the neck. An incision was made in the neck skin and the right common cervical artery (CCA), external carotid artery (ECA), internal carotid artery (ICA) and wing palatine artery isolated. A filament with a silicone tip of 2.0 cm was inserted through the incision into the lumen of the ICA and when it reached 1.8–2.0 cm it occluded the ostium of the right middle cerebral artery (MCA) to produce cerebral ischemia for 120 min; then the filament was removed to enable reperfusion. The rats underwent the Zea-Longa test (21) after reperfusion for 24 h and only those with scores of 1–3 were used for experiments. The same operation procedure was performed in rats of the sham operation group except that the filament was advanced into the ICA. The chosen area of penumbra in the cortex and striatum was as follows (Figure 1).

### Exercise Intervention

A motorized treadmill (ZH-PT/5s, Huaibei Zhenghua, Anhui) was used in the present study. Before surgery, rats in the exercise group were familiarized with treadmill running over a 3-day period (12 m/min, 10 min/day). Twenty four hour after the MCAO procedure, rats in the exercise group were trained on the treadmill (0° slope) for 30 min for 3 or 6 consecutive days. The treadmill velocity was increased every 10 min from 5 to 9 m/min and finally to 12 m/min. Rats in the MCAO and sham groups remained on the treadmill for 30 min without running.

### Evaluation of Neurological Scores

For neurological deficit assessment, all rats were scored using an 18-point modified neurological severity score (mNSS) 1, 4, and 7 days after CI-RP by an operator who had no knowledge of the treatment allocation. mNSS is comprised of motor, sensory, reflex and balance tests (22). The score was graded from 0 to 18 points, where 0 represents normality with no neurological signs and a higher score indicates a more severe neurological injury.

### Infarct Assessment

2,3,5-triphenyltetrazolium chloride (TTC) was used to estimate the volume of the brain infarct. When the last neuro-score was completed, the rats were deeply anesthetized. The brains were rapidly removed and kept at −20°C for 10 min and then sequentially sliced into 2-mm coronal sections. The sections were immersed in 2% TTC and incubated at 37°C for 30 min in the dark. Then, the brain sections were fixed in 4% paraformaldehyde for 24 h and images were recorded using a digital camera.

### Quantitative Real-Time Polymerase Chain Reaction Analysis

Total RNA was extracted from the penumbra and its concentration and purity examined using BioPhotometer plus (Eppendorf). Subsequently, RNA (1 μg) was reverse transcribed into cDNA, which was then added to a reaction system that included primers and SYBR Green. The quantity of IL-4 was determined using the Applied Biosystems (7500 Real-Time PCR Software, RRID:SCR_014596). The kits used for total RNA extraction, cDNA synthesis and rt-PCR were all obtained from Tiangen Biotech (Beijing, China). U6 (Cat#B661602-0002, Sangon, Shanghai) served as the internal control and mRNA expression was determined using the 2^−ΔΔCT^ method (23). The forward primer sequence for IL-4 was: GTACCGGGAACGGTATCCAC, which was designed based on previous studies (24). All experiments were repeated three times.

### Luminex Multiplex Assay

The supernatants from the isolated brain tissue were collected. The concentrations of cytokines including IL-4, IL-10, IL-13, IL-1a, IL-1b, IL-6, and MCP-1 were measured using the Bio-Plex Pro™ Rat Cytokine 23-Plex Assay (Cat#12005641, Bio-Rad, Hercules, CA) by Luminex technology, following the manufacturer's instructions. The cytokine concentrations were determined using a Luminex 200 instrument. The expression of proteins is given in units of pg/mg.

### Western Blotting

The extracted protein of the penumbra was homogenized in RIPA lysis buffer (Yamei, Suzhou) according to the manufacturer's instructions. The supernatant of proteins was subjected to 10% sodium dodecyl sulfate–polyacrylamide gel electrophoresis (SDS-PAGE) and transferred to nitrocellulose membranes, which were sealed with 5% skimmed milk for 2 h at room temperature. The rinsed membrane and primary antibody were incubated together overnight at 4°C, including p-JAK1 (Cat#YP0154, Immunoway, dilution 1:500), p-STAT6 (Cat#YP0256, Immunoway, dilution 1:400) and GAPDH (Cat#YM3029, Immunoway, dilution 1:5000). After 3 washes with TBST, samples were exposed for 2 h at room temperature to secondary antibodies (Immunoway, dilution 1:10,000). The bands were observed using an Odyssey Infrared Imaging System 3.0.29 (LICOR, Nebraska). All experiments were repeated three times.

### Double Immunofluorescence Staining

The brains of every group were sectioned coronally at 40 μm intervals using a freezing microtome. Slices were soaked in 0.1% Triton X-100 in PBS for 10 min and then blocked for 1 h. Subsequently, let the slices incubate overnight at 4°C in the mixed solution of rabbit anti-Iba1 (Cat# 019-19741, Wako, RRID: AB_839504, dilution 1:500) and mouse anti-CD68 (Cat# MCA341R, Bio-Rad, RRID: AB_2291300, dilution 1:50) or mouse anti-Arg-1 (Cat# sc-271430, Santa Cruz Biotechnology, RRID: AB_10648473, dilution 1:25). After rinsing 3 times with PBS the next day, incubation fluid was replaced with corresponding secondary antibodies labeled with goat anti-rabbit IgG–Alexa Fluor 555 (Cat# A-21428, Thermo Fisher Scientific, RRID: AB_2535849, dilution 1:1000) and goat anti-mouse IgG–Alexa Fluor 488 (Cat# A-11029, Thermo Fisher Scientific, RRID: AB_2534088, dilution 1:500). DAPI (Cat#G1012, Servicebio, Wuhan) counterstaining was applied to label the cell nuclei for 10 min. Images were captured using a Zeiss confocal microscope (LSM 800) and ZEN imaging systems (ZEN Digital Imaging for Light Microscopy, RRID:SCR_013672) at × 100 magnification and the number of CD68 and Arg-1 in Iba1^+^ cells in the cerebral cortex and striatum were quantified with Image-J 1.51 software (Media Cybernetics Inc. Co., Shanghai, China).

### Statistical Analysis

All data were evaluated using SPSS version 26.0 (IBM SPSS Statistics, RRID:SCR_019096) or GraphPad Prism version 8.3.0 (GraphPad Prism, RRID:SCR_002798). Mean ± standard deviation (SD) was used to describe the results. Student's *t*-test was adopted to analyze data quantitatively between 2 groups and one-way ANOVA for multiple group comparisons, followed by LSD as the *post-hoc* test. A *P* < 0.05 was considered to be a statistically significant finding.

## Results

### Treadmill Exercise Alleviated Neurological Deficits and Reduced the Infarct Volume After MCAO

**Figure 2:**
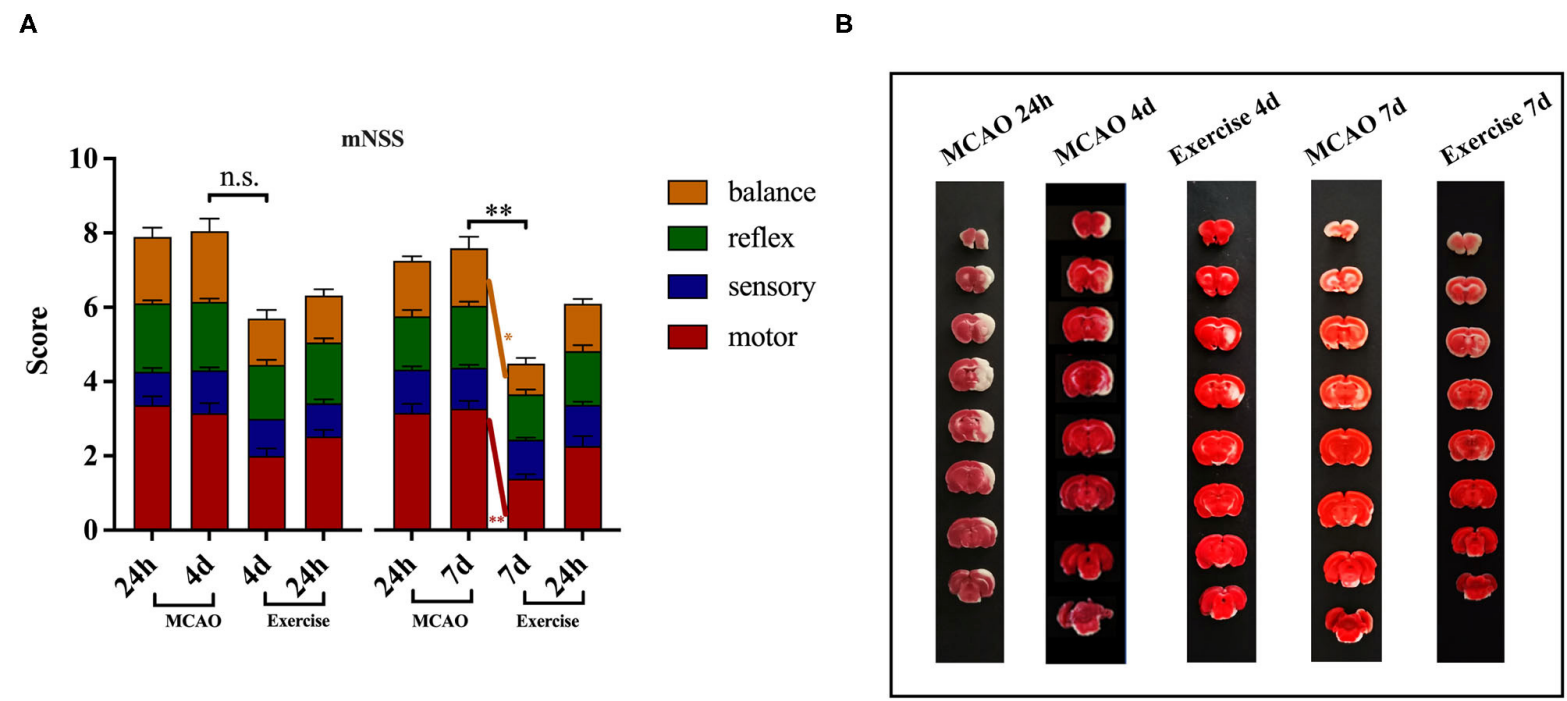
Treadmill exercise alleviated neurological deficits and reduced infarct volume after MCAO. **(A)** Behavioral functions were assessed using mNSS in the MCAO and exercise groups 24 h post-surgery before the animals were humanely killed (MCAO group: *n* = 19 for 4-day time points, *n* = 18 for 7-day time points; Exercise group: *n* = 20 for 4-day, *n* = 18 for 7-day time points). The data are presented as the mean ± SD. n.s.: *p* > 0.05, *P < 0.05, **P < 0.01 vs. MCAO group. **(B)** Representative images of brain sections (8 consecutive sections at 2.0 mm intervals from bregma) obtained after TTC staining from rats in the MCAO and exercise groups before they were humanely killed (*n* = 1 per group).

To identify the neuroprotective effects of treadmill exercise, the functional deficits and infarct volume at the indicated time points were evaluated. As shown in Figure 2A, the difference in baseline of mNSS between the MCAO and exercise groups was not remarkable. The rats treated with treadmill exercise for 4 days did not exhibit a significant decrease in neurological scores (*P* > 0.05), but showed a greater reduction at 7 days compared to the MCAO group (*P* < 0.01), especially in the motor (*P* < 0.01) and balance (*P* < 0.05) subitems. Since rats in the sham operation group had no deficit, their mNSS scores were 0, which are not reported in this paper. These data indicated that treadmill exercise was effective in promoting functional recovery after MCAO.

The TTC analysis revealed that MCAO surgery produced histologically proven damage to the cortex and striatum in the MCAO group. The increased infarct volume induced in the MCAO group appeared to be attenuated by the treadmill exercise intervention, an effect which was not quantified in the present study (Figure 2B).

### Treadmill Exercise Inhibited M1 Microglia Polarization and Promoted Microglia Polarization Toward M2 Phenotype After MCAO

**Figure 3:**
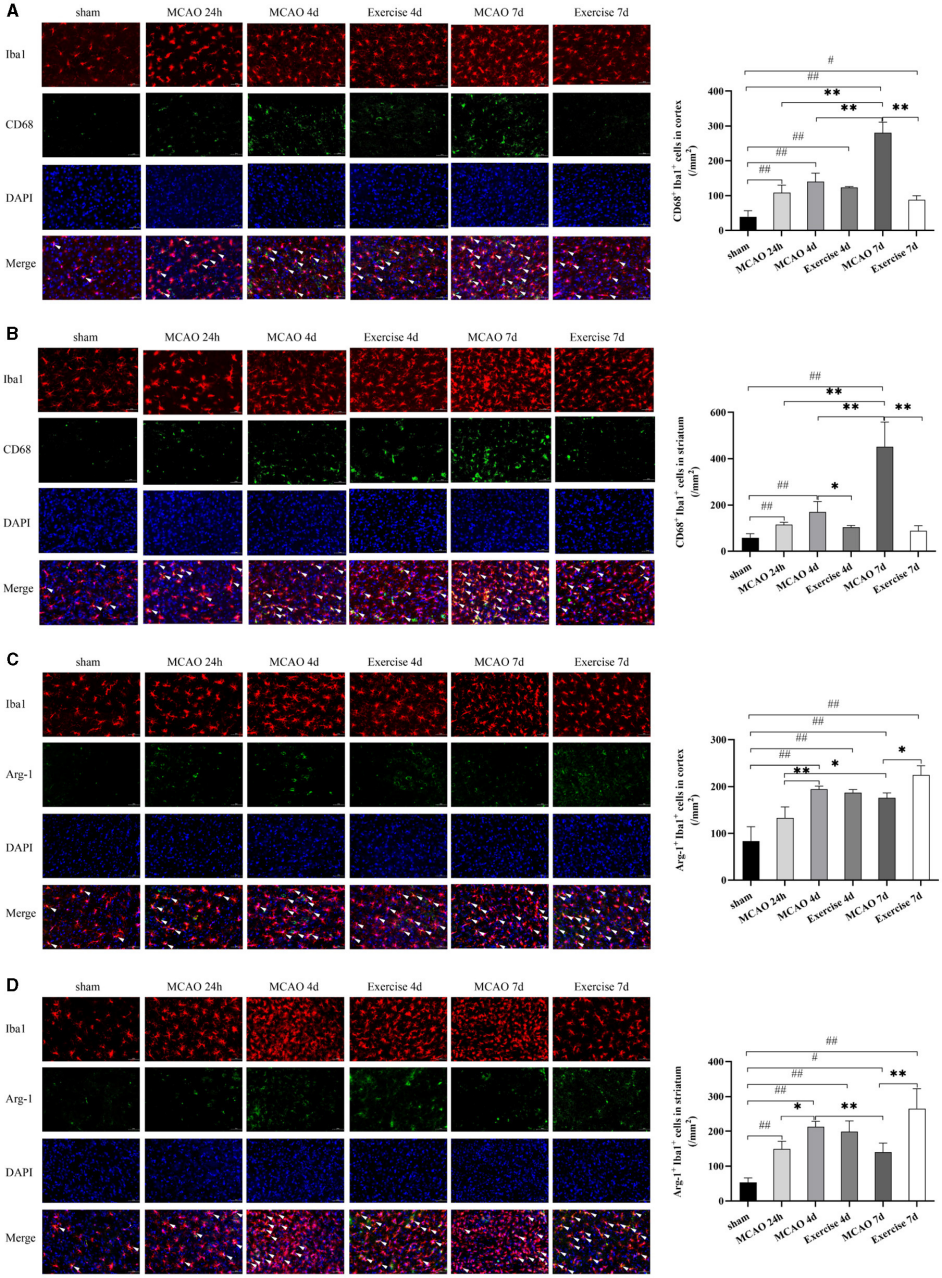
Treadmill exercise inhibited M1 microglia polarization and promoted microglia polarization toward M2 Phenotype after MCAO. Double immunostaining with Iba1 and CD68 or Arg-1 at 24 h, 4, and 7d among the sham, MCAO and exercise groups was performed in this study. **(A,B)** representative images of immunostaining with Iba1 and CD68 in the cortex **(A)** and striatum **(B)** and the corresponding histogram was shown on the right side. **(C,D)** representative images of immunostaining with Iba1 and Arg-1 in the cortex **(C)** and striatum **(D)** and the number of double-positive cells was shown on the right side. White arrows indicated the double-positive cells. Bar = 50 μm for all images, *n* = 3 per group. ^#^*P* < 0.05, ^##^*P* < 0.01 vs. sham group; *P < 0.05, **P < 0.01 vs. MCAO group.

To determine which subtype of microglia was activated in the cortex and striatum in response to MCAO and treadmill exercise, double immunostaining for microglial biomarker Iba1^+^ and M1-like marker CD68 or M2-like marker Arg-1 was performed in this study, respectively. As expected, CD68^+^ M1 microglia in the cortex (Figure 3A) and striatum (Figure 3B) was observed with a smaller number at 24 h and gradually increased over time, especially at 7 d (*P* < 0.01 for cortex, and striatum) after MCAO. Three-day (*P* < 0.05 for striatum) and 6-day (*P* < 0.01 for cortex, and striatum) of treadmill exercise significantly curbed this growing tendency. In contrast, among 24 h, 4, and 7 d after MCAO, the highest level of Arg-1^+^ M2 microglia of the cortex (Figure 3C) and striatum (Figure 3D) was observed at 4 d (*P* < 0.01 for cortex, *P* < 0.05 for striatum) and treadmill exercise lasting for 6 d raised it to a much higher level compared to the corresponding time point in the MCAO group (*P* < 0.05 for cortex, *P* < 0.01 for striatum). These data suggested that there is a fair chance that treadmill exercise suppressed inflammation by reducing M1 microglia and facilitating the expression of M2 microglia.

### Treadmill Exercise Altered the Levels of M1/M2 Markers

**Figure 4:**
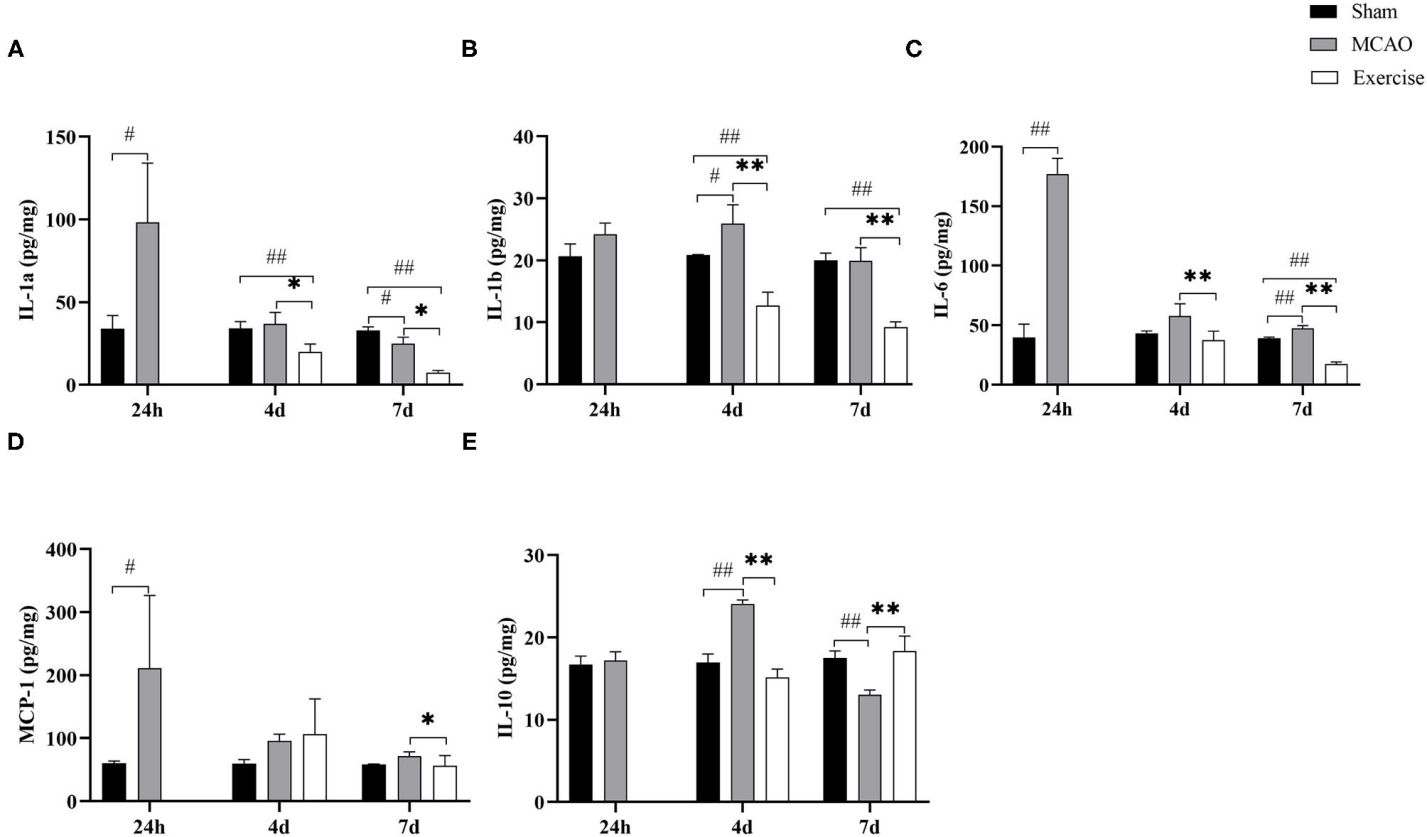
Treadmill exercise altered the levels of M1/M2 markers. The levels of M1 markers, including **(A)** IL-1a, **(B)** IL-1b, **(C)** IL-6, **(D)** MCP-1, and M2 marker **(E)** IL-10 in the penumbra at 1-, 4-, and 7-days post-surgery among the sham, MCAO and exercise groups were examined by Luminex Multiplex Assays. Data are presented as the mean SD, *n* = 3/group. ^#^*P* < 0.05, ^##^*P* < 0.01 vs. sham group; *P < 0.05, **P < 0.01 vs. MCAO group.

To explore further the effects of treadmill exercise on microglia polarization, we analyzed the expression of surface markers on M1/M2 phenotypes in the penumbra using Luminex Multiplex Assays. As shown in Figure 4, the level of M1-like markers, including IL-1a, IL-1b, IL-6, and MCP-1, and the M2-like marker IL-10 were strongly affected by the treadmill exercise intervention. IL-1a expression (Figure 4A) reached the highest level at 24 h (*P* < 0.05), then continued decreasing to the lowest level (*P* < 0.05) at 7 d after MCAO compared to the sham group. It was found that IL-1b expression (Figure 4B) was induced and elevated at 24 h, further increased and peaked at day 4 (*P* < 0.05) and then decreased at day 7 after MCAO compared to the sham group. The levels of IL-6 (Figure 4C) and MCP-1 (Figure 4D) reached their peaks at 24 h (*P* < 0.01 for IL-6, *P* < 0.05 for MCP-1) after MCAO and then rapidly declined, but still remained elevated compared to the sham group. The expression of IL-10 (Figure 4E) increased at 24 h, with a peak at 4 days (*P* < 0.01), and decreased rapidly by day 7 (*P* < 0.01) in the MCAO group. Treadmill exercise significantly reduced the protein expression of IL-1a, IL-1b, IL-6, and MCP-1 on day 4 (*P* < 0.05 for IL-1a, *P* < 0.01 for IL-1b and IL-6) and day 7 (*P* < 0.05 for IL-1a and MCP-1, *P* < 0.01 for IL-1b and IL-6), except for the MCP-1 expression at 4 d post-surgery compared to the MCAO group. In contrast to the levels of M1-like markers, the protein level of IL-10 was significantly higher only on day 7 post-surgery (*P* < 0.01) in the exercise group. These results indicated that treadmill exercise contributed to inhibiting the M1 phenotype at first and in addition then resulted in an enhancement of M2 polarization.

### Treadmill Exercise Increased the Expression of IL-4 After MCAO

**Figure 5:**
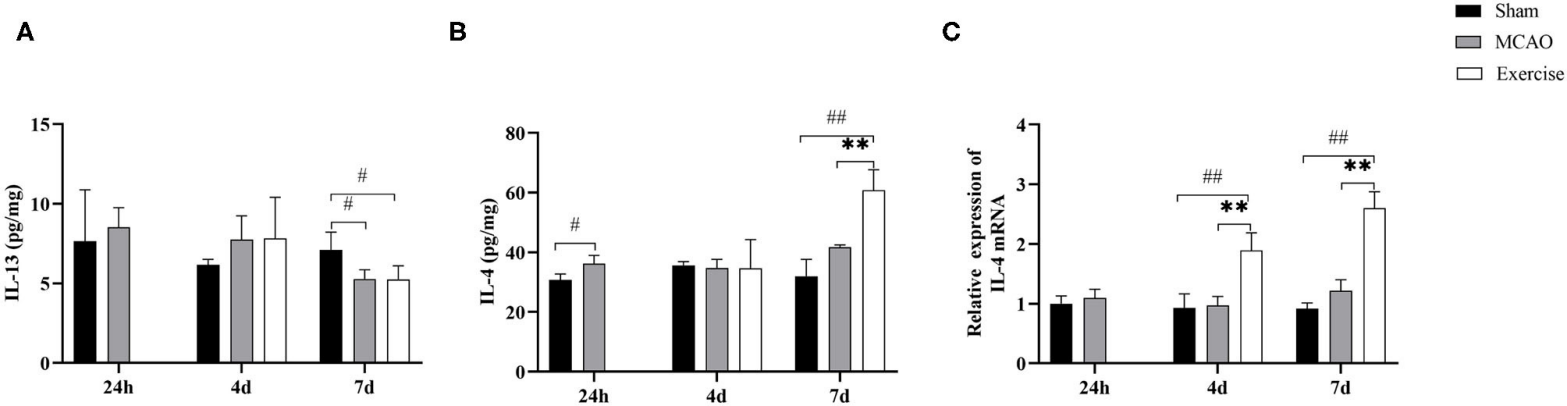
Treadmill exercise increased the expression of IL-4 after MCAO. Protein concentrations of IL-13 **(A)** and IL-4 **(B)** in the penumbra were measured by Luminex Multiplex Assays on day 1, 4, and 7 post-surgery in the sham, MCAO and exercise groups. **(C)** RNA extracts were prepared from the penumbra and mRNA analysis of IL-4 was detected by real-time PCR at the same time points in the 3 groups. Data are given as the mean ± SD. *n* = 3/group. ^#^*P* < 0.05, ^##^*P* < 0.01 vs. sham group; **P < 0.01 vs. MCAO group.

To determine whether IL-4 or IL-13 promoted M2 polarization in the penumbra after treadmill exercise, Luminex Multiplex Assays were developed to establish the protein levels of IL-13 (Figure 5A) and IL-4 (Figure 5B) at the indicated time points among the sham, MCAO and exercise groups, respectively. The results suggested that the level of IL-4 increased significantly after the 6-day treadmill exercise (*P* < 0.01) compared to the MCAO group. Subsequently, we used qRT-PCR to measure the gene level of IL-4. Data analysis clearly revealed a significant gain in the mRNA level of IL-4 in the exercise group on both day 4 (*P* < 0.01) and day 7 (*P* < 0.01) post-surgery compared to the MCAO group (Figure 5C). These findings suggested that upregulation of IL-4 signaling is involved in exercise-mediated functional recovery after MCAO.

### Treadmill Exercise Regulated Microglia Polarization Through the JAK1/STAT6 Pathway After MCAO

**Figure 6:**
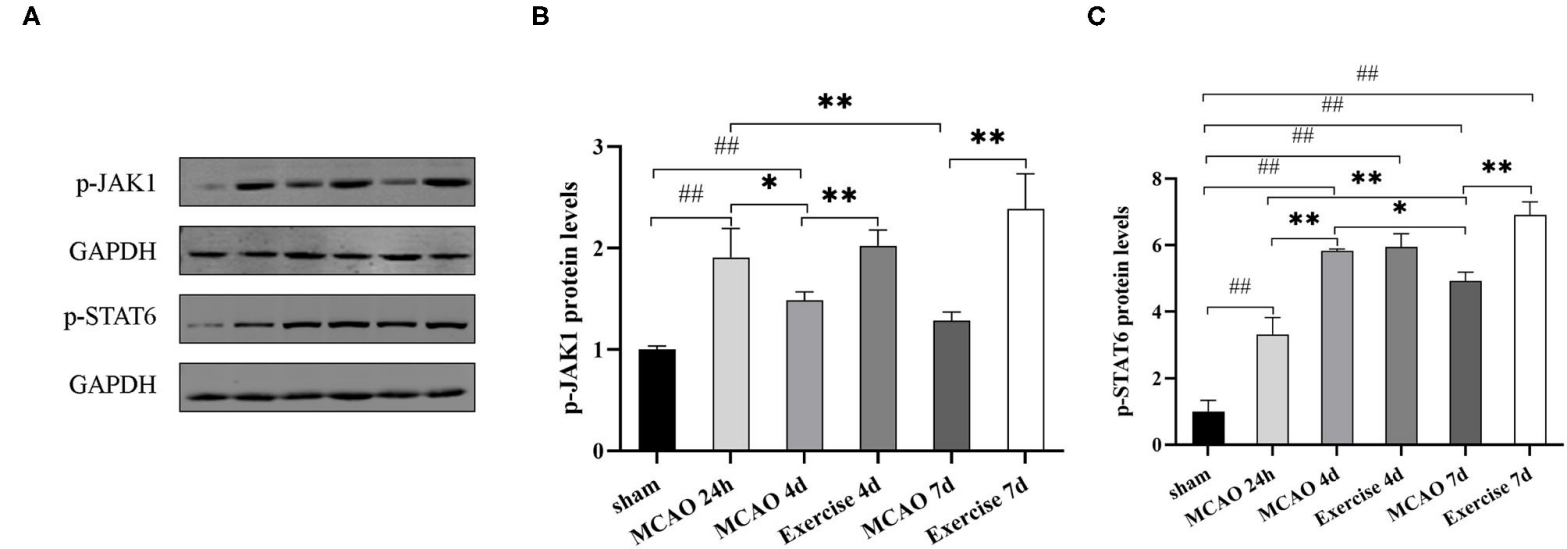
Treadmill exercise regulated microglia polarization through the JAK1/STAT6 pathway after MCAO. **(A)** The representative protein band of p-JAK1 and p-STAT6 was acquired by western blotting with the protein GAPDH serving as the control. Relative protein level of p-JAK1 **(B)** and p-STAT6 **(C)** in the penumbra on the 1st, 4th, and 7th day among the sham, MCAO and exercise groups are shown. Data are presented as the mean ± SD, *n* = 3/group. ^##^*P* < 0.01 vs. sham group; *P < 0.05, **P < 0.01 vs. MCAO group.

Treadmill exercise enhanced IL-4 expression, stimulating the upregulation of the M2 phenotype. To establish whether JAK1/STAT6 pathway activation was involved, the protein levels of p-JAK1 and p-STAT6 were detected using western blot analysis. The results showed that the p-JAK1 expression was significantly increased in the exercise vs. MCAO group on day 4 (*P* < 0.01, Figures 6A,B) and day 7 (*P* < 0.01, Figures 6A,B) post-surgery. The p-STAT6 was markedly increased at 7 d in the exercise group in comparison to the MCAO group (*P* < 0.01, Figures 6A,C). These data suggested that JAK1/STAT6 was activated after treadmill exercise intervention.

## Discussion

In the present study, we verified that treadmill exercise could exert its neuroprotective effects by downregulating the levels of the M1 microglia phenotype. To our surprise, treadmill exercise played a role in facilitating the anti-inflammatory reaction by accelerating the polarization from the M1 to M2 phenotype and by upregulating the production of the anti-inflammatory cytokine IL-4. We showed that treadmill exercise intervention significantly elevated the level of IL-4 to promote M2 polarization for brain repair and suggest that the JAK1-STAT6 pathway might be involved in mediating the IL-4 effect.

Previous reports have concluded that the onset time of therapeutic exercise did not show any benefit either within 3 h or 3 days after modeling (25). Li et al. conducted several animal studies and found that 6 h after reperfusion that inflammatory injury was exacerbated, but 24 h and 3 days stroke recovery was enhanced (26, 27). These findings are consistent with results from other studies in which exercise beginning 24 h post-surgery produced beneficial effects (28, 29). In addition, Morrison et al. showed the morphology of microglia was changed and that microglia activity was greatly decreased after 24 h of reperfusion (30). Temporal profile analysis of microglia expression and activation in ischemic animals revealed that both were significantly increased in the peri-ischemic area at 96 h and especially 7 days after reperfusion (31). Therefore, we chose 24 h after CI/RP as exercise initiation timing and day 4 and day 7 post-surgery to investigate the effects of treadmill exercise on microglia polarization and inflammatory responses.

Previous studies have shown that a long-lasting inflammatory response contributes to neurodegeneration and the aggravation of brain injury after stroke (32, 33). Exercise has been reported to induce anti-microglial activation which may effectively modulate inflammation in different diseases of the central nervous system (34–36), at least partly because of the increased secretion of anti-inflammatory factors (37–39) and a decrease in pro-inflammatory cytokines (40–42). For example, Zhang et al. found that swimming inhibited microglia activation in the hippocampus via upregulation of IL-4 after global cerebral ischemia (34). It has been reported that treadmill exercise suppressed microglia-induced neuroinflammation via CD200/CD200R signaling pathway by decreasing the level of IL-1β and TNF-α in a mouse model of MCAO (43). Although it has already been established that exercise has an effect on microglia activation, these studies did not explore the changing phenotype of microglia. An increasing body of evidence suggests that there is a dynamic microglial phenotypic polarization in the penumbra, and whether these microglia exacerbate or alleviate tissue damage is associated with their different polarization (44, 45). In agreement with these findings, we showed here that microglia express predominantly M2 markers in the penumbra between day 1 and day 4 after CI/RP, whereas the expression of M1 markers progressively increased over 7 days, suggesting a M2-to-M1 phenotype shift. Therefore, it is necessary to search for a link that would associate the neuroprotective effects of exercise to microglia polarization in the penumbra at different time points after CI/RP.

Some drugs have shown neuroprotective effects in animal models of stroke by affecting the type of microglia. For instance, melatonin treatment has been shown to ameliorate brain damage by shifting the microglia phenotype from pro-inflammatory to anti-inflammatory polarity (46). Curcumin was also found to improve functional outcomes via M1–M2 microglial switching (47). At present, M2a microglia are mainly considered to suppress inflammation (11). The M2a phenotype may be the main cell subtype involved in the processes we studied and the surface marker Arg-1 was selected as the surface marker of M2a microglia for immunofluorescence staining (48). In the present study, we found that 3- and 6-day treadmill exercise did indeed promote an M1-to-M2 phenotype shift in the penumbra, which was correlated with greater improvements in neurological scores (particularly in motor and balance) and reduced infarct volumes compared to the MCAO group. These data support the benefit of M2 induction on treadmill exercise for stroke recovery. It is worth noting that in order to induce overall beneficial effects, it is not enough to simply suppress M1 activation to block inflammation (49). Nevertheless, treadmill exercise appears to be a good therapeutic option for stroke rehabilitation.

Based on stimulation of diverse cytokines, microglia could generally polarize into the M1 or M2 phenotypes. IL-4 and IL-13 are essential effectors that polarize microglia to the M2a phenotype (50). An M2a phenotype was indeed induced after IL-4 application both *in vitro* and *in vivo* (51). In our study, the data obtained by Luminex Multiplex Assays and real-time PCR revealed a dramatic increase in IL-4 rather than IL-13 in the exercise compared to the MCAO group. In summary, treadmill exercise increased IL-4 concentrations, which was accompanied by decreased numbers of CD68^+^ M1 microglia and increased Arg-1^+^ M2 microglia. Consistent with the results of immunofluorescence staining, Luminex Assays revealed a decreased expression of M1 markers (IL-1a, IL-1b, IL-6, and MCP-1) and the increased levels of the M2 marker IL-10. Therefore, IL-4 is likely to be involved in the M2 polarization induced by treadmill exercise. IL-4 is widely recognized as a protective and pleiotropic cytokine in CI/RP, which plays an important role in inflammatory reactions by modulating microglia polarization (52, 53). Yang et al. used intracranially injection of interleukin-4 and verified that an IL-4 supplement could promote neuro-functional recovery and enhanced microglia M2 polarization *in vivo* (54). Zhao et al. reached the same conclusion from the results of *in vivo* experiments and also ascertained that IL-4 administration converted the surrounding microglia to the M2 phenotype *in vitro* (55), a finding consistent with conclusions reached after *in vi*tro experiments carried out by Ting et al. (56). These data demonstrate the importance of IL-4 in eliciting M2 polarization after CI/RP.

Binding of IL-4 to its receptor (IL-4R) elicits stimulation of intracellular signaling, such as the JAK/STAT pathway, which involves IL-4-specific transcription factor STAT6 (17). The role of STAT6 in M2 phenotype switching following IL-4 stimulation was consistently confirmed (57–59). Cai et al. discovered that knockout of STAT6 significantly reduced the positive effect on M2 microglia induction (60). Data from one study demonstrated that IL-4 could promote M2 polarization via activation of the JAK1/STAT6 pathway (61), while JAK1 inhibition would block IL-4-dependent STAT6 activation (62), suggesting that JAK1 is a key molecule downstream of IL-4 signaling. In our study, we detected the protein levels of p-JAK1 and p-STAT6 and observed that treadmill exercise markedly increased JAK1 and STAT6 phosphorylation. Taken together, it is possible that treadmill exercise elicits its favorable effects via the IL-4-JAK1/STAT6 pathway to induce M2 polarization.

Our data clearly showed that IL-4 did not greatly increase M2 induction until the 6-day treadmill exercise intervention. Recent studies also highlighted the effect of IL-4 on long-term behavioral performance after cerebral ischemia (15, 55). The evidence implied that the therapeutic effects of IL-4 polarization to an M2 phenotype is likely to accumulate over time, thereby promoting a greater degree of neurological recovery. Hence, it will be prudent to investigate further the long-term effects of treadmill exercise on IL-4 activity and the consequent alterations in microglia polarization and functional recovery; indeed the degree of exercise may be a factor leading to stress (25). Further studies should proceed more cautiously by additionally increasing the intensity and duration of exercise. Furthermore, in order to prove further the reliability of the present findings, IL-4 knockout (KO) mice or inhibitors of JAK1/STAT6 pathways should be applied in subsequent experiments for comparison.

In summary, the results support the hypothesis that treadmill exercise can alleviate inflammatory injury and promote microglia polarization to the M2 phenotype by increasing the expression of IL-4, which may activate downstream the JAK1/STAT6 pathway. Our study has identified the anti-inflammatory feature of treadmill exercise. As far as we are aware, this study is the first to show that IL-4 is an effective factor in treadmill exercise-mediated anti-inflammation, involving the induction of the M2 phenotype in microglia.

## Data Availability Statement

The original contributions presented in the study are included in the article/supplementary material, further inquiries can be directed to the corresponding authors.

## Ethics Statement

The animal study was reviewed and approved by the Ethics Committee of Shanghai Jiao Tong University.

## Author Contributions

The overall conception and supervision of this experiment was from YZ and experimental details was designed by YZ and HD. The animal experiment was carried out by JL, JW, LY, and GY and the samples' analysis was completed by JL, JW, LY, and RC. JL wrote the manuscript. JW, LY, and RC participated in the data interpretation and image acquisition. YZ revised and approved the final version. All authors read and agreed on the submitted manuscript.

## Funding

This research was supported by the National Natural Science Foundation of China (81973612) and the Science and Technology Commission of Shanghai Municipality (21ZR1459200) to YZ.

## Conflict of Interest

The authors declare that the research was conducted in the absence of any commercial or financial relationships that could be construed as a potential conflict of interest.

## Publisher's Note

All claims expressed in this article are solely those of the authors and do not necessarily represent those of their affiliated organizations, or those of the publisher, the editors and the reviewers. Any product that may be evaluated in this article, or claim that may be made by its manufacturer, is not guaranteed or endorsed by the publisher.
